# Recommendations for Designing a Digital Health Tool for Blindness Prevention Among High-Risk Diabetic Retinopathy Patients: Qualitative Focus Group Study of Adults With Diabetes

**DOI:** 10.2196/65893

**Published:** 2025-06-13

**Authors:** Akua Frimpong, Alvaro Granados, Thomas Chang, Julia Fu, Shannan G Moore, Serina Applebaum, Bolatito Adepoju, Mahima Kaur, Vignesh Hari Krishnan, Amanda Levi, Terika McCall, Kristen Harris Nwanyanwu

**Affiliations:** 1 Department of Ophthalmology and Visual Science, Yale University, 300 George Street, New Haven, CT, 06511, United States, 1 2037763539; 2 Larner College of Medicine, School of Medicine, University of Vermont, Burlington, VT, United States; 3 Department of Ophthalmology, Kaiser Permanente Bernard J. Tyson School of Medicine, Pasadena, CA, United States; 4 Division of Health Informatics, Department of Biostatistics, Yale School of Public Health, New Haven, CT, United States; 5 Department of Biomedical Informatics and Data Science, Yale School of Medicine, New Haven, CT, United States; 6 Center for Interdisciplinary Research on AIDS (CIRA), Yale School of Public Health, New Haven, CT, United States

**Keywords:** diabetes, DM, diabetes mellitus, type 2 diabetes, type 1 diabetes, diabetic retinopathy, needs assessment, blindness, mHealth, mobile health, mobile application, smartphones, applications, digital health, digital technology, digital interventions, Health disparities

## Abstract

**Background:**

Diabetic retinopathy (DR) is a leading cause of preventable blindness among working-aged adults. Black, Latine, and low-income individuals are screened less for DR, diagnosed later, treated less often, and go blind more than White individuals.

**Objective:**

This study aimed to engage members to co-design a digital health tool that is accessible, user-friendly, and culturally relevant, through a community-led research approach,.

**Methods:**

Using a qualitative approach, we conducted 4 semistructured focus group interviews with 19 individuals from the Greater New Haven area, aged 18 years or older, and diagnosed with diabetes. We transcribed and coded the focus group interviews and categorized them into themes using affinity mapping. The specific aims were to complete a comprehensive needs assessmen for the development of a community-responsive digital health tool and to increase access to information about DR screening in high-risk populations. We transcribed the focus group interviews, used rapid qualitative analysis to generate themes, and completed affinity mapping to identify content and features for a digital health tool for preventing blindness from DR.

**Results:**

We interviewed 19 individuals (68% [13/19] female, 47% [9/19] Black, 26% [5/19]) Hispanic) in 4 focus groups. Over 80% (15/19) had access to smart devices, including smartphones (17/19, 89%), smartwatches (4/19, 21%), computers (14/19, 74%), and tablets (11/19, 58%). Many participants had access to multiple devices (17/19, 89%). Participants self-reported hemoglobin A1c (mean hemoglobin A1c 6.77, SD 1.93) and age (mean age 58.79, SD 19.54). Education levels among participants varied. Almost half of all the participants (9/19, 47%) completed some college, a little less than a quarter (4/19, 21%) achieved a high school diploma or general education development certificate, and a little less than a quarter (4/19, 21%) completed less than a high school equivalent of education. Household income levels across nearly all participants (14/19, 74%) were below US $50,000, but household size data were not collected. Participants reported extensive experience with diabetes or prediabetes (mean years with diabetes or prediabetes 17.06, SD 17.53). The themes obtained from coding focus group interviews included the mental toll of diabetes, peer support like accountability and local community events, education about diabetes management, barriers to DR screening like long wait times for appointments or cost of medications, and diet-related topics like how to find cost-effective healthy food

**Conclusions:**

DR is one of the leading causes of blindness, and many treatments exist. Despite the existence of treatments, historically marginalized populations experience poor health outcomes, including blindness. Our community-based approach aids in the creation of a culturally responsive digital health tool.

## Introduction

Diabetic retinopathy (DR) is a leading cause of vision loss and blindness in the United States [1]. Routine eye examinations facilitate early detection and prevent vision loss, as DR is asymptomatic in early stages [2]. The prevalence of DR among people with diabetes was approximately 26% in 2021 [3]. Despite its high prevalence, less than 50% of individuals with diabetes in the United States undergo the recommended annual DR screening [4].

Screening rates vary widely among sociodemographic groups, and most of the groups that remain unscreened and untreated are historically marginalized communities, specifically Black, Latine, and low-income communities [5,6]. These communities are less likely to be aware of DR and experience from reduced engagement in eye care [7]. The barriers to care are complex and include interplays between insurance coverage, knowledge gaps regarding DR and the value of eye health screening [8].

In recent years, developers created mobile apps that focus on delivering diabetes-related education and reducing barriers to health care engagement [9]. However, the majority of studied mobile app interventions lack a culturally informed approach for Black, Latine, and low-income populations, which reduces their generalizability to these communities [10-12]. Studies show that cultural adaptations of health care interventions increase their effectiveness [13,14]. Structural racism, systemic oppression, and ongoing underrepresentation in health research contribute to delays and diminished access to health technology for traditionally underserved populations [15-17]. Patients at high risk of blindness from DR necessitate a culturally sensitive digital health tool that prioritizes their needs. One study tailored the design of a digital tool specifically for low-income Arabic individuals living in Israel, however, their intervention provided only diabetes-related dietary knowledge [18]. There is a clear and urgent need for content and features to improve DR screening rates and provide comprehensive diabetes-related support.

Individuals with diabetes often describe the mental toll of living with diabetes. Adverse psychological experiences are documented in the literature. There is a significant stigma surrounding diabetes, which leads to feelings of shame, guilt, or failure, especially for public management of the disease, like administering insulin [19]. Studies suggest that negative emotions play indirect causal roles, and themselves are outcomes of poor self-management of diabetes [20-22]. Fisher et al [23] showed that even at subclinical levels below the mild-to-moderate depression diagnosis threshold, depressive symptoms and emotional distress are linked to worse diabetes self-management. A systematic review found that psychological conditions, including depression, anxiety disorders, eating disorders, and other severe mental illnesses, are consistently associated with poorer self-management of diabetes in adults [24]. They further noted sources of anxiety related to diabetes, including hypoglycemia and fear of complications and invasive procedures [24]. It is documented in the literature that positive pathways of living with diabetes include support from family, friends, and other informal social networks [25]. Social support networks are often facilitated through the use of digital technology, which provides immediacy in social connections and fosters a sense of community [26]. Our study aims to lay the groundwork for the community-led design of a digital health tool that prioritizes community building to mitigate the mental toll of diabetes.

While various technologies exist that address the disparate needs of individuals with diabetes, there does not exist a single, comprehensive, digital health tool that integrates all the necessary facets. The purpose of this study is to identify the content, features, and key considerations for the community-driven design of a digital health tool to empower and promote marginalized groups’ engagement in diabetic eye care and reduce disparities in preventable blindness.

## Methods

### Study Design

We included focus groups based on the Consolidated Framework for Implementation Research. All focus group sessions were audio recorded and transcribed by a professional service, Atomic Scribe. Subsequently, the focus group interview transcripts were coded and categorized into themes used for affinity mapping.

### Recruitment of Participants

The research team (JF, TC, BA, MK, AF, TM, and KN) and members of a Community Advisory Board actively recruited potential participants from the New Haven community. We posted flyers throughout the New Haven community including in the Yale Eye Center, the Dana Eye Center, Yale affiliated primary care physician (PCP) and endocrinologist’s offices, recreational centers, community restaurants, churches, and recruited by word-of-mouth. We also reached out to individuals who previously expressed interest in participating in studies at the Sight-Saving Engagement and Evaluation in New Haven (SEEN) Lab. For inclusion in the study, individuals needed to (1) be at least 18 years old, (2) have a diagnosis of diabetes, (3) speak English fluently, and (4) have reliable transportation to attend the in-person focus group.

We screened potential participants via telephone using a screening questionnaire. Each focus group session lasted approximately 90 minutes and included a dinner that accommodated participant food allergies and diet preferences.

### Ethical Considerations

The institutional review board (IRB) at Yale School of Medicine approved the study and all participants gave informed consent (IRB #2000034710). This study adhered to the Declaration of Helsinki. Participants were consented before the beginning of any research activities, including the baseline assessment, by the research assistants (JF, TC, AF, BA, and MK), who were trained in the process. The Yale IRB reviewed all materials for ethical standards, and compliance. Privacy and confidentiality standards were maintained through the use of deidentified data for sharing with data analysts, and Protected Health Information was kept digitally on a private, encrypted server, while paper records containing Protected Health Information were kept in a secure, locked area that only provided access to the research team. Participants who completed the study were each compensated with a gift card containing $50 US dollars.

### Data Collection

The research team developed an interview guide Multimedia Appendix 1. We developed the interview guide using a community-based approach by consulting with an advisory board comprised of members from the community. We collaborated with members of our Community Advisory Board to refine the questions, ensuring they accurately addressed specific community needs and contexts (Multimedia Appendix 2). The focus group interview guide included questions about the following topics: (1) participants’ experiences with diabetes (2) their eye health awareness, (3) their technology usage, and (4) their preferences for features in a digital tool to learn about eye health and screening. The team refined the interview guide during data collection to include emergent themes and improve question clarity. This iterative process allowed the team to adapt to participant feedback and incorporate prompting questions to elicit comprehensive responses.

### Focus Group

We conducted semistructured focus group interviews in 2 community spaces. Results from a study by Guest et al [27] revealed that more than 80% of all themes are discoverable within two to three focus groups, and 90% of themes could be discovered within 3 to 6 focus groups. Therefore, 4 focus group sessions were conducted. Each session was capped at 6 participants to allow for all participants to fully engage in the discussions. We ensured the setting was conducive to discussion, collaboration, and confidentiality. AF (medical student) moderated each session, and multiple notetakers (VR, KN, TM, JF, TC, MK, and BA) attended different sessions to take notes on body language, nonverbal cues, and emerging themes. The moderator and notetakers debriefed after each interview. For the first 30 minutes of the focus group session, notetakers and the moderator ensured that participants completed the demographic surveys and signed the consent forms after demonstrating full understanding of them. We informed participants that all information would be deidentified and used solely for study purposes. The sessions were recorded with an audio recorder and we used a backup recorder on the computer. At the end of the sessions, each participant was compensated with a US $50 gift card. After each focus group, the moderator and notetaker debriefed. We sent all audio recordings to Atomic Scribe for transcription and redaction of identifying information to ensure participant confidentiality. We securely stored all data in an encrypted server.

### Data Processing and Analysis

The analytic team was comprised of members from the SEEN Lab (AF, TC, JF, KN, VH, AG, and SA) and the Consumer Health Informatics Lab (TM, BA, MK, AL). Team members included a vitreoretinal surgeon, retina fellow, consumer health informatics expert, user experience and user interface designer, medical students, graduate students with advanced degrees in business, health sciences, and public health and researchers with extensive experiences working with marginalized populations.

#### Rapid Analysis

We (AF, TC, JF, MK, BA, KN,and TM) conducted a rapid analysis of each focus group session transcript to efficiently process data and share findings with the broader study team, to provide insights for our user-centered design process [28-32]. Analytic team members developed and used a rapid review analysis template to organize and interpret the interview data systematically [28,29]. The template guided the documentation of the analyzer’s initial impressions, detailed accounts of participants’ experiences with diabetes care, their use of technology in health care and eye care, and the analyzers’ summary thoughts on each interview. The initial rapid analyses for each focus group session was led by TM within 1 week after each focus group session. The team recorded emerging themes and quotes.

#### Interpretation Sessions (Affinity Mapping Analysis of the Focus Group Interviews)

Following the initial analysis, each notetaker presented common themes from focus group transcripts to the broader research team. TM led interpretation sessions using affinity mapping, a type of cluster analysis, in which the team coded the interviews and categorized the comments into themes [33-37]. Interviewers met in 7 sessions between February 2024 and April 2024 to transform raw data from the transcripts and initial rapid analysis into Miro, an online tool for visual collaboration via creating and grouping online “sticky notes.” Each sticky note represented a subtheme identified by a single interviewer, coded and colored to correspond to that interviewer. Each interpretation session included the research team (TM, AF, TC, JF, KN, VH, AG, SA, BA, and MK, SA, AG, and AL). The focus was on extracting the most pertinent information from each interview. The interviewers provided important details from information gleaned from the interview and their interactions with potential users. Key points, observations, and challenges in the design process were recorded. Finally, the modelers generated contextual design models to visualize the main data extracted from the focus group sessions (eg, affinity diagram). In the final session, the research team prioritized and categorized the important features and ideas that participants suggested, based on their impact and ease of implementation. After the last session, the study results were presented to the advisory board for reflection and feedback to refine the must have features list.

## Results

### Demographics and Baseline Characteristics

Thematic saturation was achieved with 4 focus groups (n=19, 2‐6 participants each focus group session). We recruited 24 individuals, of whom 19 attended one focus group session. Focus group sessions were approximately 90 minutes long (60 min devoted to the semistructured interviews). The study participants included a diverse demographic, with 68% (13/19) female, 47% (9/19) identifying as Black, 26% (5/19) as Hispanic, and 11% (2/19) as indigenous. Over 80% (15/19) of participants had access to smart devices, defined as either smartphones, smartwatches, computers, and tablets.

Most participants, over 70% (13/19), disclosed annual household incomes below US $50,000. Hemoglobin A_1c_ (HbA_1c_) was self-reported (mean 6.77, SD 1.93). Ages ranged from 35 years old to 72 years old (mean age 58.79, SD 19.54). Approximately half (10/19, 52%) of participants did not pursue higher education (neither partially nor completed a college degree). Over one quarter (5/19, >26%) of participants reported having a diagnosis of DR. Of note, one group consisted of all women, and another group included 3 participants with physical disabilities.

During the initial rapid qualitative analysis, the research team (KN, BA, AF, MK, TC, and VH) presented the main takeaways from the focus group sessions. The main findings highlighted the app should include: (1) education and resources on diabetes, (2) ability to communicate with providers, (3) options to personalize the user experience, (4) features that facilitate peer support, and (5) components that address emotional and mental stress associated with diabetes diagnosis and management. Supporting quotes for the main themes are presented in Table 1.

**Table 1.** Supporting quotes for the needs and priorities of participants to inform the development of a digital health tool to improve management of diabetes and diabetic retinopathy.

Themes	Examples	
Resources		
Low-cost medications and treatments	“My problem is paying for the medications. One of the pens that I use costs US $60, and I use three of them.” [Speaker 1, Group 4]	
Affordable healthy food	“Because it’s hard to buy food.... You got to buy certain foods to keep your diabetes in check. Fruit and vegetables, they’re up sky-high.” [Speaker 3, Group 2]	
Access to providers	“Sometimes it’s hard to get in there. And my appointment is at 2, and I don’t be seen until 4:30... If I’m on time, you should be on time.” [Speaker 1, Group 2]	
Difficulty securing eye care	“I was just going to do it [go to the appointment] in January. And the other thing is that I moved, so from [Redacted] County to [Redacted] County. So, I had to find all these new doctors.” [Speaker 2, Group 4] “There is a lot of people that really need help. There’s a lot of people that really have bad diabetes. They not even have insurance.... And nobody will help them. They not even know where to go.” [Speaker 2, Group 2]	
Exercises	“The one that could test your blood sugar. One that could tell you if you’ve drank enough water. And if you’re too high, maybe it’ll recommend perhaps get another 2000 steps in or something.” [Speaker 1, Group 1] “I’m in this chair. I really can’t exercise, because I can barely stand up. All I do, all I know how to do is get up in the morning and check my sugars and stick myself.” [Speaker 1, Group 2]	
Eye care	“Again, I knew, from my mom and because I watched her, that deterioration. She wasn’t always blind, but I watched that about I would say 10 or 15 years.” [Speaker 2, Group 4] “I found out when I had double vision, that’s when I found out it’ll affect your eyes. Too little, too late, but at least I’m on it now.” [Speaker 5, Group 3]	
Transportation to appointments	“Sometimes it’s hard getting around here in the wintertime, and I don’t go [to the appointment].” [Speaker 1, Group 2]	
Healthy recipes	“On Facebook I get one of the [recipe] things in my feed, but they are not diabetic-friendly. They’re just the opposite.” [Speaker 1, Group 4] “I wonder if a menu planning app would be useful…it tells you what the sugar, what the fiber, what the carbs, all those things are.” [Speaker 6, Group 3]	
Education	“I think it’s education, too. Even my father having [diabetes], they didn’t know why he was so angry all the time, fighting and acting out. They didn’t know his blood sugars affected his mood.” [Speaker 2, Group 1] “I don’t understand it. Somebody, break it down for me. Just don’t everybody keep repeating the same thing to me, I know that a friend of mine has lost his two legs.” [Speaker 2, Group 4]	
Communication with providers		
Suggested queries for health literacy	“FAQs^a^, because things come up that you didn’t think of… If I could just open up an app and say, ‘Answer the question’, and it would take me to some kind of explanation or answer, that would be useful.” [Speaker 6, Group 3] “[I would like to know] What complications could arise if you don’t get your sugar under control.” [Speaker 5, Group 3]	
MyChart link	"I would say with the MyChart, I like it because whether I’m in Raleigh, North Carolina, no matter where I’m at, when I go to the emergency room and I couldn’t talk, they were able to pull up my MyChart and understand what was going on and what was wrong with me.” [Speaker 3, Group 1]	
Personalized user experience		
Notifications	“Notifications? Like, yeah, if you’re going high, [or] you’re going low. But something that could also just balance everything and let you know, “this is where you normally at, and now you’re off by this or that.” [Speaker 3, Group 2] “But I don’t need an app telling me, ‘All right, you got to do this now,’ for somebody [like me] that’s been living with it for so long.” [Speaker 1, Group 4]	
Incentives	“Maybe a point system too with the app and incentive[s]. Maybe after so many points or so, logging in or taking care of yourself or something, [you get] a free meal, [to a] different restaurant. [Laughter].” [Speaker 2, Group 1]	
Facilitating peer support		
Accountability	“Sometimes I find the need for someone to hold me accountable. ‘What did you eat today? Why did you do that?’” [Speaker 5, Group 3] “My family would embarrass me. They’d be like, ‘Why are you eating that? Aren’t you diabetic?’"[Speaker 4, Group 3]	
Local DM^b^ events/groups (virtual and in person)	“Maybe some kind of communication board, ‘I have your type of diabetes in your age group.’ I don’t have a support network. Might be nice to connect with someone online, just to check in with each other.” [Speaker 5, Group 2]	
Mental impact		
Isolation	“I get so depressed. I didn’t even want to walk outside. Almost all my family died from diabetes. So, I start[ed] thinking about me, my kids and my grandkids, and I make [sic] the decision to try to control my sugar.” [Speaker 2, Group 2]	
Polypharmacy	“And so now I am on two different insulin[s] that I take. They’re thinking about putting me on Metformin again, so that’ll be three. And I am so sick of it. It adds to my depression.” [Speaker 4, Group 1]	

a FAQs: frequently asked questions.

b DM: diabetes mellitus.

### Resources

Participants expressed a desire for diabetes-specific diet advice, particularly regarding specific food choices and how much to eat as well as finding ways to exercise. Some participants highlighted a need for easy recipes and meal plans for cooking at home, incorporating nutrition information and comprehensive recommendations. When dining out, participants said they would benefit from education regarding what is considered fast food, and what take-out options are better choices. Participants expressed frustration at the obscurity of food labels and wanted education on reading them to better understand their food’s nutritional content. Participants understood the importance of exercising but found it difficult to exercise, especially when it came to feeling motivated. Participants expressed wanting resources for all types of skill levels in exercising and having a group event to motivate them to exercise.

But that’s my whole journey with this diabetes…I’m trying to get my weight down because I realize if my weight is down and I exercise more, [and] I’m moving more, it will go away. I think I can live with a prediabetes.

[Speaker 4, Group 3]

So, it took a compilation of all the foods, So I think that would be great too, just to have an app that would automatically do that,” [Speaker 3, Group 1]. “Something that’ll help you with a diet,”

[Speaker 3, Group 2].

“Like maybe a meal planner.

[Speaker 5, Group 2]

### Education

Participants sought to know what the effects of diabetes are on their health. Many participants desired informative content in the app regarding diabetes. For example, participants requested lists of “do’s and don’ts for people with diabetes” and “frequently asked questions.” Some participants also requested comprehensive information regarding the complications and side effects of diabetes, results from new diabetes-related research, and information about novel treatments and their effects.

When you’re not compliant and you’re out of control, your sex drive drops. I thought I was [just] getting older… those interpersonal things that nobody wants to talk about… Let’s everybody bring that forward.

[Speaker 3, Group 1]

But a lot of trial and error came from, as I said, watching my mom, watching things with her. And it changed a lot, because I remember going to the hospital and she had that orange. They were teaching her how to stick herself, give herself insulin, which was having an orange, and she was injecting under the skin on the orange. They don’t do that anymore.

[Speaker 2, Group 4]

How [blood sugar] needs to be checked frequently. How [my vision] could blur. About eating different things could help my vision. And I said, ‘Well, wow, I didn’t know that.’

[Speaker 1, Group 3]

### Eye Health

When it came to understanding how diabetes affects the eyes, some participants shared that they were informed about the risk of vision problems and the importance of eye care at the time of their diagnosis. In contrast, others expressed that they were not told that diabetes can lead to blindness. Some learned about eye care through personal experiences or from family and friends, while others were informed by their health care providers. Many recognized the benefits of regular eye exams, with most having had an exam within the past year to a few months ago, despite some difficulty scheduling appointments. The group had a positive attitude toward diabetes eye care, although experiences varied. Participants also expressed a desire to better understand how diabetes affects the eyes.

So, I had really, really good—have really good vision. But at one point when my diabetes was uncontrolled… I couldn’t see the license plate right in front of me. Like everything was blurry and starry at night. So, I couldn’t even drive. [My brother] had to drive for me at one point, having my own vehicle.

[Speaker 2, Group 1]

When I first became diabetic and I found out, like I said, my sugar was like 740, and I remember everything being blurry. I was so used to being in shape, doing all these sports… it really blindsided me. It’s like getting ear-holed by a wide receiver.

[Speaker 3, Group 1]

### Barriers to Health Care

Participants spoke of the barriers that they face accessing health care. Long wait times to scheduling an appointment with a physician was a common topic, as was limited access to health care. Participants also reported challenges with the sheer amount of medications to take and their associated cost, availability, and side effects. Several participants reported financial barriers, citing the high overall cost of care. Many participants were well-versed in the need to make lifestyle changes but reported barriers to implementing them including transportation, the increasing cost of healthy foods and comparatively high accessibility of fast food.

…sometimes it’s hard to go to get an eye exam because they charge so much. It’s expensive when you go get an eye exam. And plus, those testers…some of it’s out [of] pocket… So that’s the main hurdle right there, is try[ing] to afford that test.

[Speaker 3, Group 2]

My mother was a diabetic. My father was a diabetic. Her mother was a diabetic. So, it ran in the family. I still don’t get this. I can’t do certain things. I’m in this chair. I really can’t exercise, because I can barely stand up. All I do, all I know how to do is get up in the morning and check my sugars and stick myself.

[Speaker 1, Group 2]

### Access to Health Information

Participants expressed a desire for a user-friendly digital health tool that is informative without being overwhelming. They suggested features such as a point reward system, customization options, and streamlined content. A key priority was improving communication with healthcare providers, with participants highlighting the need for supplementary materials and guidance to facilitate conversations with doctors. They also showed interest in opportunities to participate in research studies and clinical trials. Some participants spoke about having a digital tool that makes records easy to access, like MyChart.

Participants discussed their preferences for receiving information, including push notifications from the app. They valued notifications about new treatments, support groups, upcoming doctor’s appointments, medication reminders, exercise reminders, blood glucose, and blood pressure tracking.

So maybe an app that would say, [if] you start to have blurred vision or something like that…what you should or shouldn’t do. And maybe what kind of foods you should eat that will make your eyesight better.

[Speaker 4, Group 3]

### Support and Engagement

Participants consistently identified family as a source of support. Some participants had older family members with diabetes, so they grew up witnessing the experience of diabetes, forming a unique perspective. Others spoke of friends and community as a resource. Support for these individuals came from community health centers and health care providers. In contrast, other participants described a need for stronger support due to lack of knowledge or commitment from their existing family or community. Participants voiced that they would like to have a platform to build community.

[My friend]'ll say, ‘Okay, did you do your shots, your pills and your drugs?’ So she reminds me all the time of it, but… she tempts me, “Let’s go have sprinkles.“

[Speaker 1, Group 4]

You were asking about support. she has MyChart, and I have a Hero…A Hero is really good. It dispenses my medications for me. But that’s all on her phone as well, my daughter. So, she sees that as well. She says, “Mommy, why haven’t you taken such and such? You know you’ve been taking it late.

[Speaker 2, Group 4]

### Mental Health Impact

Participants spoke of the emotional toll that their chronic disease has had on them. They described feelings of depression, denial, embarrassment, and psychosocial struggles. They spoke of the emotional journey that living with diabetes engenders and seeking answers to how diabetes complications can affect their mental capacity.

I’ve been a diabetic for 20 years. It’s a nightmare. I remember the day that they told me that I was a full-blown diabetic. I cried my eyeballs, and I went into denial. . . I wouldn’t take care of myself. They started me with Metformin. . . and I wouldn’t take the pill. I would eat whatever I wanted to eat. . . wouldn’t exercise until I got sick. I got really sick, [my blood sugar] went up to 589. I had to be hospitalized.

[Speaker 4, Group 1]

I believe I was . . .19, 20 when it came, because I had kind of quit football because I gave up because it gave up on me. I was depressed, so my weight went up to like 375. I was a size 56. And one day, I just could not fix my craving for something to drink. . . my grandmother took me to the hospital. . . I found out my sugar was like 740. . .they told me if I didn’t lose at least 150 pounds, I was going to die. So, it took me about two and a half years to get my weight down. It wasn’t easy. I had to stop eating a 48-box of Snickers. I had to. . .serve myself less. . . I won’t go back to [eating] trash to get it, because I had 5700 calories and I’m starving.

[Speaker 3, Group 1]

**Figure 1.**
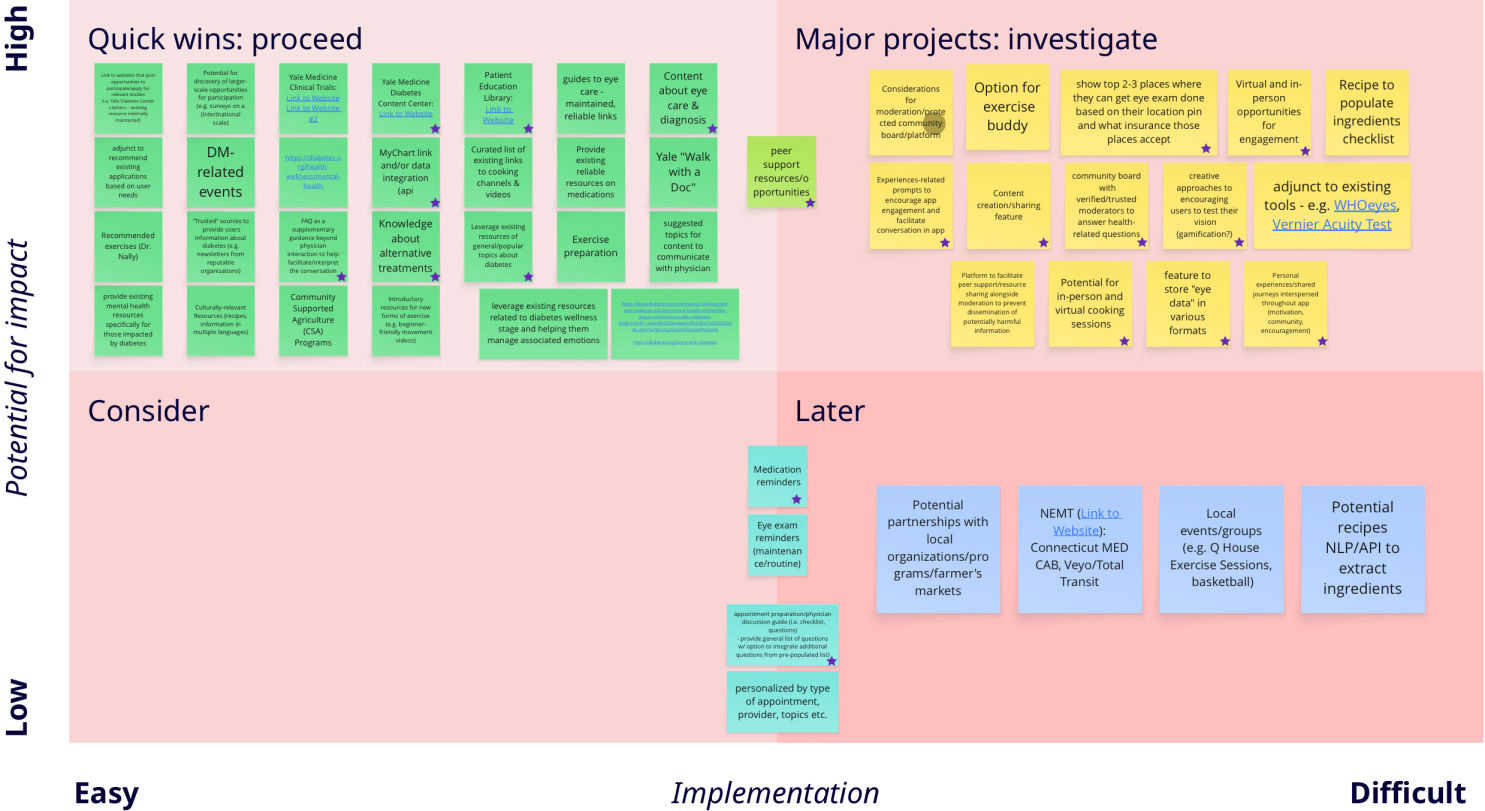
Prioritization and categorization of features and ideas for digital health tool design based on impact and ease of implementation.

Figure 1 presents features organized by impact potential and implementation difficulty. The development of the digital health tool prioritized the must-have features, located to the upper left quadrant. These were considered the most important to the participants and feasible for the research team to execute. The digital health tool will include reliable content about eye care, diabetes, and related health topics, addressing the need for trustworthy information about diagnosis, medications, and treatments. To further promote holistic wellness, it will offer resources for exercise, cooking videos, mental health, and general wellness, and other functions to provide, promote, and support overall well-being. Features will inform users of local events, particularly those related to diabetes, offer resources for clinical trials, established programs, and integrate relevant existing applications for managing patient health data. The advisory board showed consensus on the must-have features. Additional recommendations included adding testimonials and tutorials to the “Frequently Asked Questions” section, and establishing partnerships with local businesses, advocacy organizations, and public works departments, such as the city health department and the National Association for the Advancement of Colored People, to address social needs. In addition, the advisory board highlighted the value of community groups and discussion forums for individuals with diabetes.

## Discussion

### Principal Findings

Our findings highlight the need for a culturally responsive digital health tool for individuals with diabetes. This tool will incorporate lifestyle resources, informal support platforms, and electronic health record access while maintaining a well-calibrated notification system. These findings align with previous research on developing smartphone apps for diabetes management, emphasizing the importance of usability, patient-centered resources, and seamless integration with existing health care infrastructures. The consistency with other studies highlights the critical role of tailored digital tools in enhancing diabetes care and patient engagement [9,10,12]. The need for a patient-centered digital health tool that provides access to resources like guided communication with health care providers, eye health, and diabetes education. Key features include personalized user experiences, customizable notifications, peer support, and community-building platforms. Consistent with existing literature, our study confirms that patients with chronic conditions like diabetes prefer technology that supports online community engagement and self-management resources, including exercise routines, healthy recipes, and telehealth options, to enhance their overall health management [38].

### Technological Tools for Diabetes Management

Studies show that technological innovations for diabetes management, particularly through mobile devices, can improve health outcomes [10,39]. Low-cost, user-friendly digital tools that centralize information and functionality can facilitate patient autonomy and improve access to health guidance. These technologies can further inform medication management, blood glucose monitoring and subsequent communication with health care professionals [40]. A recent benchmark study evaluated the usability and content of available mobile apps for people with diabetes [41]. In their systematic evaluation, most apps had good usability and potential to facilitate diabetes management. Common app features included nutritional and dietary planning, monitoring glycemic control, and scheduling health appointments. Content areas that were less well represented included mental health, education on diabetes complications, and content aimed at individuals with disabilities. No apps existed with content related to DR or resulting visual impairment [41]. While many digital tools do exist, there are significant content gaps among available resources. There is a widespread need for an app that provides reliable information and guidance for people with diabetes who want to prevent or manage their visual impairment resulting from DR.

In addition to content within an app, the design of an app impacts its success. Specifically, the screen or display size, color, material, menu structure, user-friendliness, accessibility, and simplified operations can influence user adoption and use, particularly among older adults [42,43]. Therefore, when designing interventions or applications designed for older adults with diabetes, health care providers or app developers should focus on aesthetic features that consider the physical, sensory, and cognitive changes experienced by older adults, such as impaired vision, slow hand movement, and decreased response time [44-46].

### Mental Health

The impact of chronic disease on mental health is well-documented. Individuals living with type 1 or type 2 diabetes are at heightened risk for depression, anxiety, and eating disorders [47,48]. Many people with chronic diseases, including diabetes, use the internet to access psychological treatments [49-51]. Baldwin et al [52] explored the use of the web-based electronic mental health program, myCompass. MyCompass is both clinically effective and cost-efficient in expanding access to mental health services [50] and has previously demonstrated efficacy in reducing depressive symptoms and diabetes-related distress in individuals with type 2 diabetes. However, a notable limitation of MyCompass is that being primarily a mental health support program, it does not provide support targeting specific diabetes needs [52]. More recent studies incorporate diabetes-specific content [53] alongside existing depression treatments and cognitive behavior therapy [54]. Participants reported significant improvements in health behaviors, such as healthier eating habits, better health monitoring, and incremental progress in overall outcomes [52]. With increasing recognition of the importance of mental health in diabetes care [55], our study acknowledges the need to address mental health in the management of diabetes in the design of our proposed digital health tool.

### Community Space

Several studies emphasize the critical role of social support in diabetes management [56-58]. Toobert and Glasgow [59] identified it as a vital factor in self-care adherence, while Marquez et al [60] noted its impact on physical activity and weight loss. Shayeghian et al [61] linked social support to better blood glucose control, and Pereira et al [62] highlighted its importance for self-care behaviors, such as glucose monitoring. Diabetes online communities have emerged as valuable platforms for peer support, fostering communication, improving disease management, and enhancing psychosocial well-being [63].

There are few studies on the role of online peer support in diabetes care. One study used digital observation, applying ethnographic techniques to study interactions on social media and the web to analyze hundreds of diabetes-related posts on platforms like Facebook (Meta), Twitter (X), and YouTube (Google), identifying 6 core themes—humor, diabetic pride, technology use, tips and tricks, community building, and venting [64]. The findings emphasize the importance of online platforms in fostering camaraderie, peer mentorship, and shared experiences often overlooked by health are systems. In addition, it has been shown that integrating mental health services and peer support is beneficial and critical for positive short- and long-term diabetes outcomes [64-66]. Our digital tool aims to replicate these positive outcomes by creating a supportive environment for patients to connect with each other and share their experiences, thereby enhancing their overall care.

### Health Literacy

A significant concern for participants was their lack of knowledge about how diabetes could affect them. These gaps included understanding the full range of symptoms, complications, predisposing factors, and psychological impacts of diabetes. Research shows that individuals without diabetes self-management education are less likely to engage in preventative care, leading to worse outcomes [67]. Limited health literacy is recognized as a significant barrier to improving health outcomes. Individuals with low health literacy may have less knowledge about diabetes, have less control of blood glucose levels, engage in less self-management behaviors, and incur higher health care costs [68]. One study demonstrated a direct correlation between health literacy and several key factors in diabetes management, including self-monitoring, nutrition and exercise habits, diabetes knowledge, self-care practices, and social support [69]. Another study recommended that targeted educational and behavioral interventions are crucial in addition to health care service strategies, particularly for older adults and individuals with less formal education [10,65,66].

With the rise of social media use, patients with diabetes are more likely to seek information using technology to help manage their condition [70]. In one study, nearly half of participants sought self-management information online, with dietary planning being the most common focus [71,72]. The term eHealth literacy refers to the critical skills needed to effectively use technology for seeking, accessing, and understanding health information from electronic sources [73,74]. Previous research demonstrates a significant association between eHealth literacy and health behaviors. Lower eHealth literacy is linked to poor diabetes self-management and worse health outcomes, particularly among older adults with diabetes [75,76].

Incorporating design preferences of intended users into digital health tools may increase participant engagement and empower them to manage their health better [13,14]. This includes trust-centered design elements such as reliable content on eye care, diabetes, social support, and healthy lifestyles [13,14]. Studies suggest that digital tools should be specifically designed to address diabetes-related concerns [58,77,78].

These findings align with the discussed topics that participants acknowledged and the importance of incorporating eHealth literacy and social support into digital health tools.

### Eye Health

Some existing apps related to DR include the National Eye Institute’s virtual reality app, which simulates vision loss, and the RetinaRisk (Retina Risk) app, which calculates personalized risk for diabetic retinopathy. However, few apps focus extensively on DR content or address visual impairment resulting from the disease. Our participants expressed a strong desire for this type of content. Our digital health tool will fulfill this gap by incorporating trusted resources for education, research opportunities, community events, and mental health support tailored to eye health and diabetes care.

### Strengths and Limitations

Although this study has many strengths and advantages, there are several limitations and disadvantages when conducting focus group discussions as part of the methodology. Some challenges include limited control over data collection, the influence of group dynamics on participants’ responses, and the difficulty in generalizing findings to a larger population.

Focus groups can sometimes include silent or dominant participants, which may influence the conversation dynamics and impact data collection [79]. To address this, the moderator (AF) and observers (VH, KN, TM, JF, TC, MK, and BA) implemented strategies to ensure that all participants had an opportunity to share their views, such as redirecting the discussion to stay on topic, rephrasing questions to allow wider range of responses, and seeking clarification to encourage the expression of diverse viewpoints and perspectives.

Our focus groups had homogeneous backgrounds (diabetes status and geographic location), while also having heterogeneous traits (age differences, gender, race, and ethnicity) that ensured a diversity of perspectives and greater depth of analysis into the challenges they faced [80]. Although participants in this study shared similar opinions and perspectives, their experiences may not be generalizable to the broader population of individuals with diabetes mellitus, particularly given the geographical restriction of recruitment of the participants, which limits the national representativeness of the sample [81].

Participant recruitment included the participants self-reporting their health measures such as current hemoglobin A_1c_. This form of providing information can introduce a potential source of error or bias due to self-reporting that cannot be verified [82]. The SEEN tool has the potential to address this limitation by allowing participants to input their information or data more accurately for recall and reporting. For instance, the tool may be interoperable with glucose monitors, allowing participants to integrate their glucose data into the tool, thereby minimizing the likelihood of self-report errors.

This study did not collect household size data, which limits our ability to infer whether individuals met the criteria for poverty. However, the experience of multiple individuals manifesting through direct quotes from them is that medication costs, food costs, and other financial barriers impact access to eye care and screening. Whether or not any participants have experienced financial difficulty with managing their diabetes, their statements point to the universality of financial barriers to health care equity.

Despite these limitations, the study results yielded key insights that will inform the design team on what community needs to prioritize in the development of the digital health tool. Furthermore, our community-based approach lifts the voices of participants who are historically under-represented in medical research, and empowers them with lasting educational benefits and other resources to facilitate their management of DR.

### Future Directions

The main findings of this study serve as a guide for the design priorities for a digital health tool tailored to individuals with diabetes or DR.

The next steps, which is currently underway, will be to develop an initial prototype of the digital health tool through 3 phases of production: design, development, and evaluation of the digital health tool. During the design phase, we will create detailed user journey maps, develop health education content for the tool, and develop low-fidelity wireframes and interactive high-fidelity prototypes. All of this will be worked in collaboration with the community members to incorporate feedback and ensure the tool is intuitive and user-friendly. After the design phase, the team will begin the development phase to build the digital health tool by collaborating with developers to build the tool. Finally, the final phase will be to conduct usability testing and implement a pilot study to collect quantitative and qualitative data to test the prototype with application users in order to ensure the tool meets the community’s needs.

Future studies can expand the scope and the focus groups. The focus groups can consist of more diverse populations, including different age groups, genders, socioeconomic statuses, and cultural backgrounds, to gather a wide range of perspectives. Researchers could also include participants from various geographic locations as well to understand regional differences in digital health needs and preferences.

### Conclusions

Using a user-centered design approach, when developing digital health tools, has the potential to significantly improve adverse health outcomes for individuals living with diabetes through culturally relevant and personalized interventions. The community-centered approach not only empowers participants from communities which are often unheard to give their voice to health care research, but will also educate them through an educational component, including connecting the participants to resources to help them manage DR.

## Supplementary material

10.2196/65893 Multimedia Appendix 1 Focus Group Session Questions.

10.2196/65893 Multimedia Appendix 2 Digital Health Tool Pre-Survey/Questionnaire.

## Abbreviations

DR: diabetic retinopathy
HbA_1c_: Hemoglobin A_1c_
IRB: institutional review board
NAACP: National Association for the Advancement of Colored People
PCP: primary care physician
SEEN: Sight-Saving Engagement and Evaluation in New Haven
