# Supplementary material for: Recommendations for Designing a Digital Health Tool for Blindness Prevention Among High-Risk Diabetic Retinopathy Patients: Qualitative Focus Group Study of Adults With Diabetes
Source: JMIR Form Res. 2025 Jun 13;9:e65893. doi: 10.2196/65893 (PMC12180678; doi:10.2196/65893)
Supplement: Multimedia Appendix 2 [file formative-v9-e65893-s002.docx]

## **Appendix B:** Digital Health Tool Pre-Survey/Questionnaire

1. What is your age?
2. What is your gender?
3. What is your race?
4. What is your ethnicity (Hispanic, non-Hispanic)?
5. How long ago were you diagnosed with diabetes? (Months, years)
6. What was your most recent A1C? When?
7. Has a doctor ever told you that you have diabetic retinopathy, or that diabetes has affected your eyes?
8. Do you have a smart phone?
9. Do you use a smartwatch (Apple Watch, Google pixel watch, Samsung galaxy watch)?
10. Do you have access to a computer?
11. Do you have access to a tablet?
12. What is the highest level of education you have attained? (Some high school, High school diploma, some college, college degree, graduate degree)
13. What is your approximate household income?
